# The ability of oriental magpies (*Pica serica*) to solve baited multiple-string problems

**DOI:** 10.7717/peerj.9200

**Published:** 2020-05-19

**Authors:** Lin Wang, Jinxin Guo, Heng jiu Tian, Jinling Sui

**Affiliations:** 1 School of Ecology and Nature Conservation, Beijing Forestry University, Beijing, China; 2 Beijing Wildlife Rescue Center, Beijing, China

**Keywords:** Oriental magpies, Pica serica, Multiple-string problems, Complex cognition, Side bias strategy, Corvids

## Abstract

**Background:**

Baited multiple-string problems are commonly used in avian laboratory studies to evaluate complex cognition. Several bird species possess the ability to use a string pull for obtaining food.

**Methods:**

We initially tested and trained 11 magpies to determine whether the oriental magpie (*Pica sericia*) possesses the ability to solve baited multiple-string problems. Eight of the birds obtained the bait by pulling, and were selected for formal multiple-string tasks in the second stage. Second stage tests were divided into seven tasks based on string configurations.

**Results:**

Only two magpies were able to solve two tasks: one solved the task of parallel strings, and the other solved the task of slanted strings with the bait farther from the middle point between the two strings and selected the short string in the task of long-short strings. When faced with more difficult tasks (i.e., the task of slanted strings with the bait closer to the middle point between the two strings, the task with two crossing strings, and the task of continuity and discontinuity), the birds initially observed the tasks and chose instead to adopt simpler strategies based on the proximity principle, side bias strategies and trial-and-error learning. Our results indicate that the oriental magpie had a partial understanding of the principle of multiple-string problems but adopted simpler strategies.

## Introduction

Decades of studies have shown that complex cognitive abilities are not unique to primates and other large mammals; birds also possess a similar learning capacity (Emery & Clayton, 2004a). The anatomy of bird brains differs greatly from those of mammals (e.g., the forebrain of birds does not have a layered structure) (Medina & Reiner, 2000; Zorina & Obozova, 2011). Large-brained corvids reportedly possess forebrain neuron counts equal or greater to primates with much larger brains. The large numbers of neurons concentrated in high densities in the forebrain may substantially contribute to the neural basis of avian intelligence (Olkowicz et al., 2016). Corvids and parrots have consistently demonstrated more sophisticated qualitative and quantitative intellectual skills than other birds (Emery, 2004, 2006; Emery & Clayton, 2004b), and are similar to primates in some aspects of social ecology, neurobiology, and life history (Emery, 2006; Seed, Emery & Clayton, 2009).

Most animals, including birds, are limited in their ability to operate tools so multiple-string problems are used to test complex cognitive abilities (Vince, 1961; Bagotskaya, Smirnova & Zorina, 2012). The 1970s saw a shift toward studying developmental and sensorimotor aspects of cognition under the influence of Piaget (Jacobs & Osvath, 2015). In such tests, food (the bait) is tied to one end of a string and the animal can gain access to the food only by pulling the string (Heinrich, 1995). The debate on cognition of string-pulling tasks is ongoing. It remains unclear whether and to what extent cognitive understanding contributes to successful performance on string-pulling problems. Various multiple-string tasks can test different cognitive mechanisms. When an individual subject is faced the task of slanted strings with the bait closer to the middle point between the two strings, the task with two crossing strings, and the task with a right-angled turn on the longer baited string, the probability of wrong choice is relatively high if the subject adopts the strategy of proximity or side bias, but if the subject chooses to adopt the strategy of trial and error the probability of wrong choice will be greatly reduced (Jacobs & Osvath, 2015; Hofmann, Cheke & Clayton, 2016; Manrique et al., 2017).

Many studies have reported on the abilities of mammals and birds to solve multiple-string tasks. Mammalian studies have focused on non-human primates such as infant chimpanzees (*Pan troglodytes*) and cottontop tamarins (*Saguinus oedipus*) (Hauser, Kralik & Bottomahan, 1999; Hauser et al., 2002; Spinozzi & Potí, 1993) or carnivores such as domestic cats (*Felis catus*) and dogs (*Canis lupus familiaris*) (Riemer et al., 2014; Osthaus, Lea & Slater, 2005; Whitt et al., 2009). Avian research has primarily focused on certain birds in the family Corvidae in the order Passeriformes, or on birds in the order Psittaciformes (Werdenich & Huber, 2006). There are relatively few studies of this kind for other animals.

In the study of bird string-pulling tasks, strings are usually oriented either in a horizontal or a vertical fashion. A horizontal string can be reeled in with a single pull, whereas a vertical string requires better coordination and multiple-step motor planning with coherent movements (Werdenich & Huber, 2006; Jacobs & Osvath, 2015). A planar arrangement for multiple-string problems may reduce the need for animals to coordinate their movements under the aforementioned conditions. These approaches allow for testing various types of multiple-string tasks without the need for additional suspended items to hold the strings (Bagotskaya, Smirnova & Zorina, 2012; Manrique et al., 2017).

The majority of multiple-string task cognition experiments in Corvidae have been conducted with members of the genus *Corvus*. However, a few cognitive studies have reported on *Pica* species and indicate that the magpie (*Pica pica*) can remember the location of stored items (Clayton, 1998) and recognize themselves in a mirror (Prior, Schwarz & Güntürkün, 2008). The black billed magpie (*Pica hudsonia*) shows a superior ability to learn abstract concepts, like other jays (e.g., *Garrulus glandarius*) (Magnotti et al., 2016). There are few researchers who have studied the string-pulling tasks of distant relatives of Corvids, such as western scrub-jays (*Aphelocoma californica*; Hofmann, Cheke & Clayton, 2016) and green jays (*Cyanocorax yncas*; Manrique et al., 2017), and there are no reports on the multiple-string task cognition of *Pica* birds. The oriental magpie is a medium size bird of corvidae, widely distributed throughout China, and was revised from subspecies (*Pica pica serica*) to species (*Pica serica*) based on DNA sequencing results (Song et al., 2018). Our study is the first of its kind to determine whether the oriental magpie has the ability to solve multiple-string problems and obtain food. The aim of this study was to investigate what strategies are used by oriental magpie when confronted with various multiple-string problems. While many works on the cognition of string-pulling tasks had focused on the genus *Corvus*, little is known on the abilities of other corvids in these tasks. Assessing the performance of a more distantly related genus, the genus *Pica*, will be informative as to the distribution of complex cognition across the Corvidae.

## Materials and Methods

### Animals and installation of the experimental device

Eleven rescued oriental magpies (*Pica serica*; six males and five females, all 1 to 2 years old) from the Beijing Wildlife Rescue Center were used in this study (Table 1), which was conducted from October 2016 to August 2017. The sex of the birds was identified according to our previous report (Wang et al., 2019) and individuals were marked with colored leg rings to assist in their identification. The birds free-ranged in a 600 × 400 × 460-cm indoor aviary at Beijing Forestry University (Beijing, China) for one and a half months prior to the start of experimentation. Fresh food and water were freely available, and their diet consisted of insects, shrimps, vegetables, fruits, nuts, seeds, omnivorous bird compound feed, poultry compound vitamins and an occasional feeding of beef. The composition of the food remained unchanged during the 24 h before onset of the experiment, with the exception of insects. The food supply was withheld from the subjects while the experiment was being conducted and food (the bait) could be obtained only by pulling the string.

**Table 1:** Test results of the individual oriental magpies during the first stage of experiment.

No.	Sex	Testing	Learning	Training
P1	♂	Fail	Fail	Pass
P2	♂	Fail	Pass	–
P3	♂	Fail	Pass	–
P4	♀	Fail	Pass	–
P5	♀	Fail	Pass	–
P6	♀	Pass	–	–
P7	♂	Fail	Fail	Pass
P8	♀	Fail	Fail	Pass

**Figure 1:**
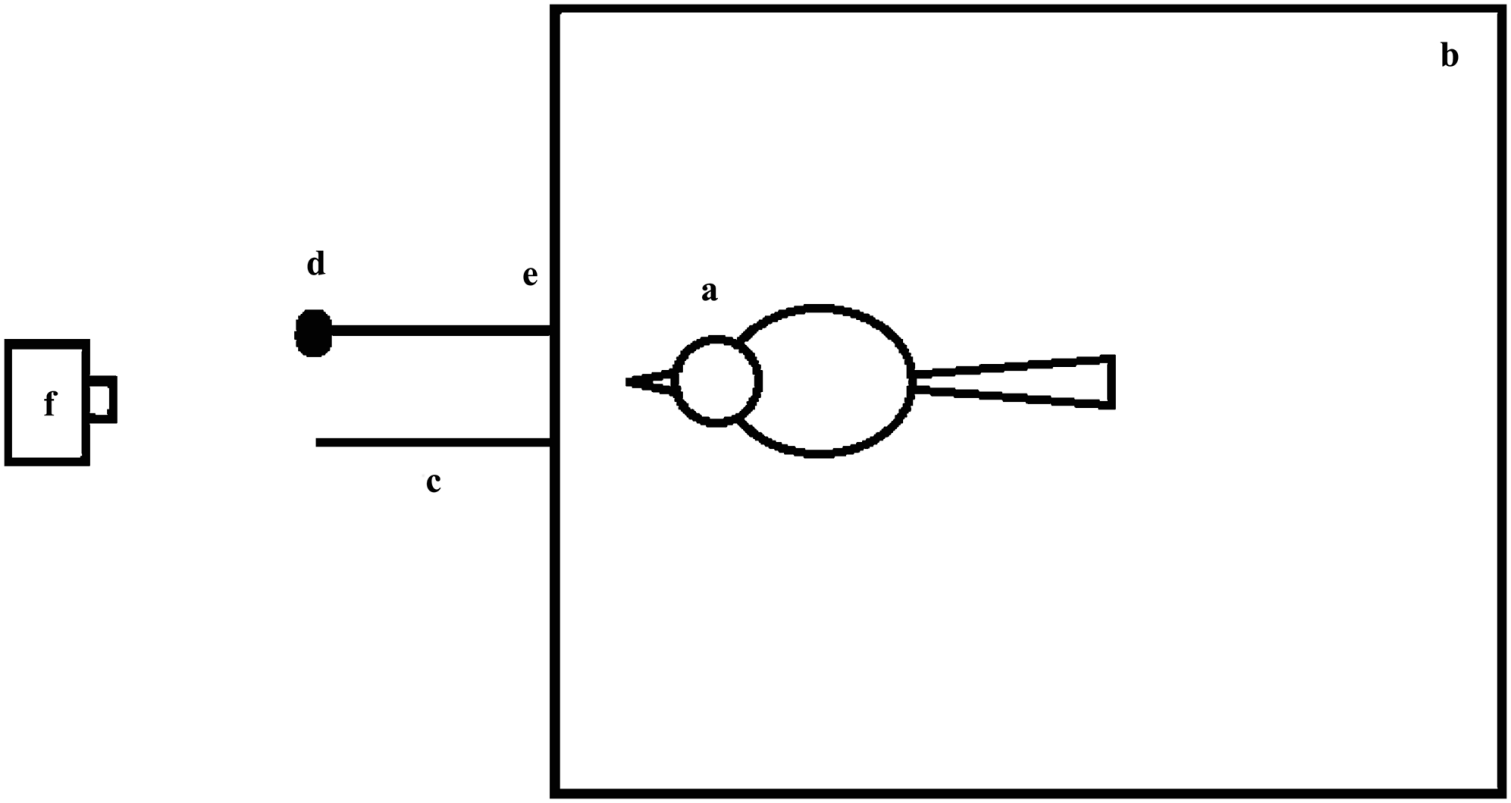
Top view of the experimental device. (A) Bird; (B) cage; (C) string; (D) bait at the distal end of the string relative to the bird under test; (E) points at which the string was tied to one side of the bottom of the cage (i.e., the end of the string near the bird); (F) video camera. The strings were located outside of the cage, and the two connecting points (13-cm apart) between the strings and one side of the bottom of the cage were located on both sides of the midpoint of the cage. The camera was placed directly in front of the two strings, and the lens was aimed at the midpoint of the bottom of the cage and covered the entire cage.

The experiment was conducted in an 80 × 80 × 50-cm cage (Fig. 1), which was placed in an adjacent indoor aviary. One end of a string was connected to the cage so that it could not be swallowed by the birds. Both the string and the bait were outside of the cage so that the birds could see the bait but could not obtain it directly. The two strings connected to the cage was 0.1 cm in diameter, the distance between the two connecting ends tying the string to the cage wall was 13 cm, and the lengths of the strings and the manner of placement varied among the experimental groups. Larvae of *Zophobas atratus* were used as bait and the bait was connected to one of the strings; the other string was not baited (Fig. 1). The entire experiment was recorded using a digital video camera (Eos M3 (WH); Canon, Tokyo, Japan) placed in front of the strings (Fig. 1).

Each bird spent an hour per day in the experimental cage for 30 days prior to the start of the experiment to become familiar with the experimental environment and to provide for their comfort during subsequent experimentation. Individual birds were placed inside the cage at the onset of the experiment and when the bird first pulled the string it was regarded as consent to proceed and the experiment began. Magpies could not leave the experimental cage until the end of the test. The subjects could view the operations when the baits were updated, and they have 30 s to observe the operation in advance. Once the experimenter left the lab, the subjects made their choice and the test was timed. The experimenter left the room prior to the start of the experiment to avoid influencing the feeding behavior. The recorded video files were analyzed after the conclusion of the experiment.

### Experimental protocols

Subjects were transferred to the test cage to begin the experiments (Fig. 1). The experiments were conducted in two stages: the first stage, that is, the pre-testing, learning and training stage and the second stage, that is, the formal string-pulling task stage, which was applied to subjects that passed the first stage of the experimentation.

#### The first stage: pre-testing, learning and training of oriental magpies for the string-pulling task

**Figure 2:**
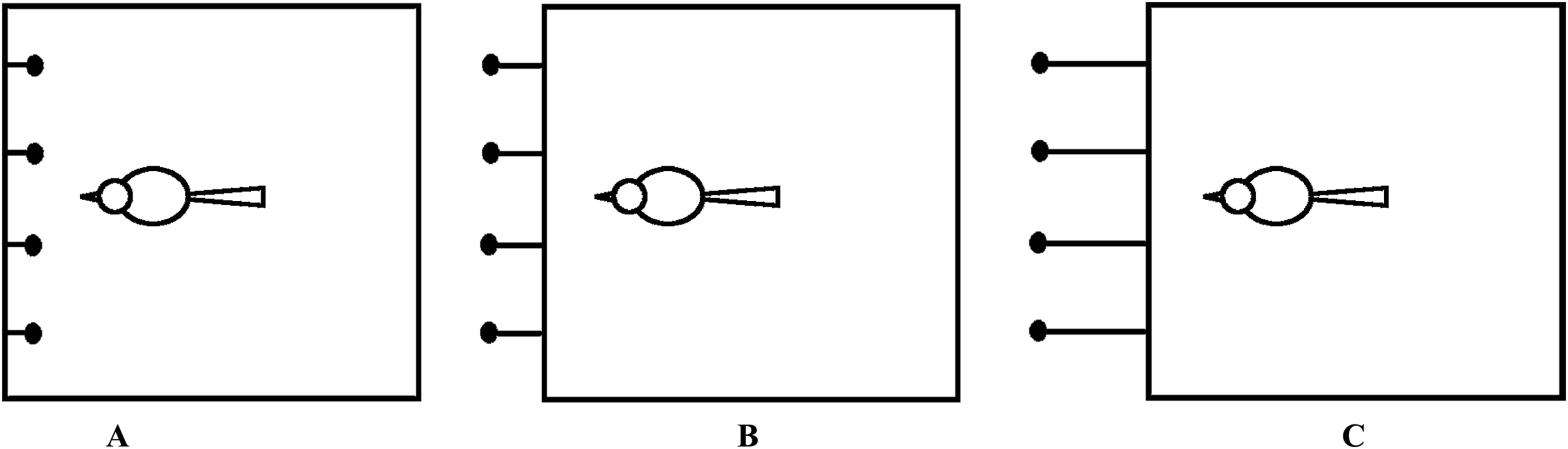
Top views of the test devices for the first stage of the experiments for string-pulling testing, learning and training of oriental magpies. (A) Baits attached to the ends of the four strings within the cage; (B) baits attached to the ends of the four strings outside the cage and gradually pulled away from the cage until the subject could not directly reach the baits unless by pulling the strings; (C) baits attached to the ends of the four strings outside the cage with a distance of 25 cm.

In the pre-testing phase, oriental magpies that had not been exposed to the string-pulling task were given a pulling task without learning or training to determine whether they would pull the string spontaneously to get bait. The end of each string was tied to bait and there was no empty string ends. The baits were placed outside the cage so that the bird could only get food by pulling the string (Fig. 2C). Each bird was tested four times in 1 day. In order to reduce the influence of frequent appearances of experimenters on the magpie, four strings, each with a bait, were put in front of the magpie at the same time, and the pre-testing was conducted only once for each subject, lasting for 20 min. Once the bird pulled the string and reached the bait, it was determined that the bird had the potential to pull strings to get bait.

Individual birds that did not know how to string and those did know how to string to obtain the baits were paired and placed in two adjacent cages (Fig. 2C) in which they could see, but not access, the string and bait tied to each other’s cage. Birds that did not pull a string to obtain the bait were given 20 min to observe the behavior of the other group to see if they would acquire this ability. During the 20 min observation of learning string-pulling behavior, the birds involved in the learning faced four strings connected to the baits at the same time as in the pre-testing process. The completion of four string-pulls was regarded as successful learning for the individual birds. The strings in this learning phase were all connected with bait with no empty ends and this process allowed more birds to participate in subsequent experiments.

Training refers to the string-pulling exercise for individuals that did not pass pre-testing and did not succeed in subsequent learning exercises. The training phase was divided into two steps: first, the bait was attached to the bottom edge of the cage so that the oriental magpies could eat the bait directly (Fig. 2A); the bait was then attached to the end of the string and was gradually pulled away from the bird at the bottom edge of the cage (Figs. 2B and 2C). In this second step, the oriental magpies could not reach the bait directly and this was done to observe whether they could get food by pulling the string. Training was only conducted once. In the training stage, the magpies would face four strings at the same time in three ways as shown in Figs. 2A, 2B and 2C, and they would have 20 min to observe and get the bait in each way. All trainees passed the first step and the oriental magpies that completed the second training step were selected for further experiments. All of the strings in the training phase were connected to bait.

#### The second stage: tests of understanding for multiple-string problems by oriental magpies

**Figure 3:**
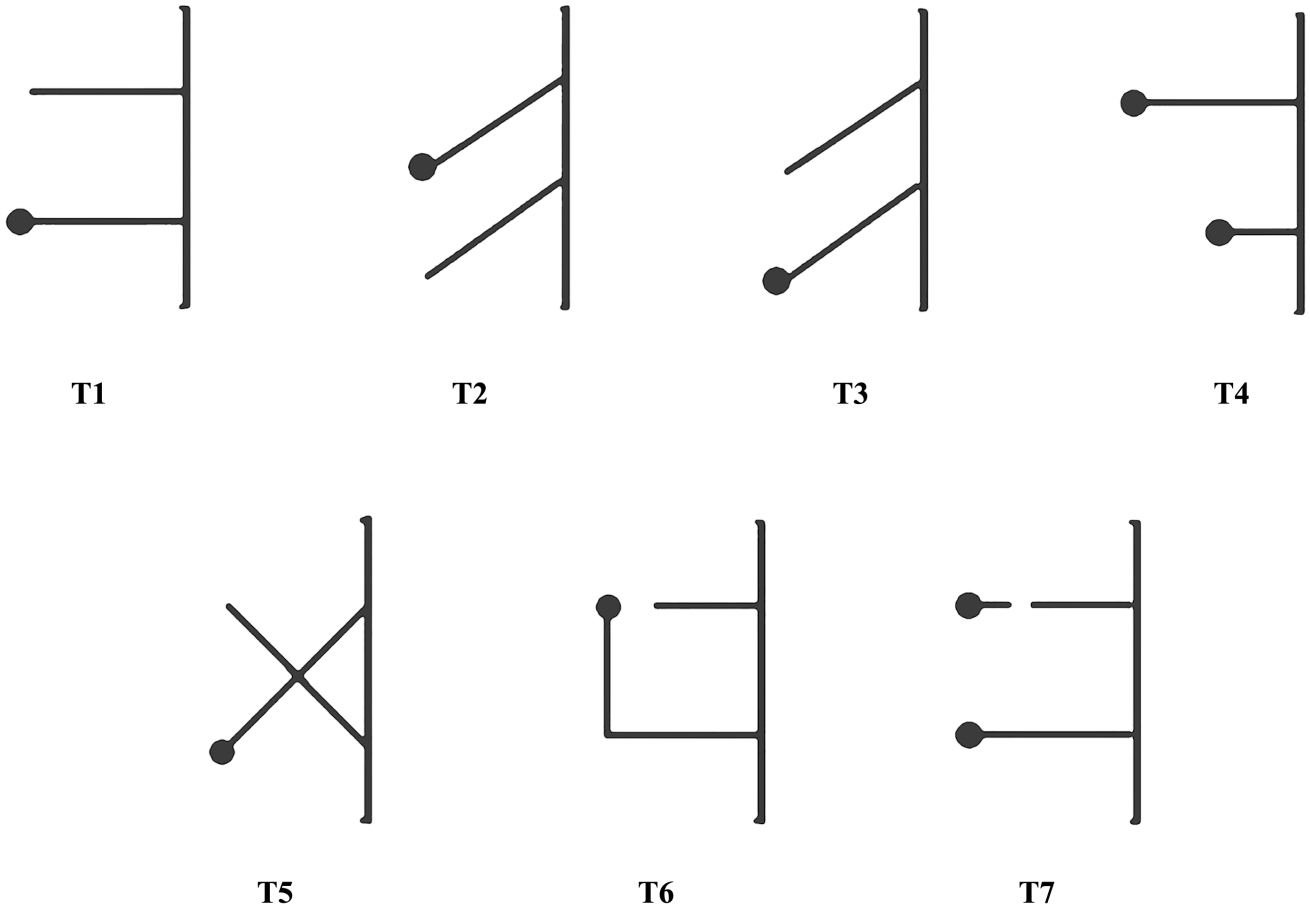
Schemes of the seven tasks. T1–T7, Tasks 1–7. T1–T4 were simpler, with T5–T7 being relatively more complex.

The second stage of experimentation consisted of seven tasks with different multiple-string problems (referred as T1–T7; Fig. 3). There were 30 trials for each task and each trial was recorded and immediately ended when a magpie pulled the string within 15 min, regardless of whether it pulled the string with or without bait at the end, which was considered success or failure, respectively. We stipulated that the first string touched by a magpie was the string chosen by the individual. Our magpies could only test the next multiple-string problem after finishing the previous tested task, which usually took at least 3 days. The order of testing proceeded from simple to complex, as shown in Fig. 3. The difficulty of multiple-string problem was determined by whether the two strings cross, change direction, or break. The position of the bait was random to avoid memory. And in order to avoid fatigue-related error, each bird performed only one of the seven tasks per day with no more than 10 trials conducted daily.

The two strings were placed on the ground outside of one side of the cage for all seven tasks (Figs. 1 and 3). One end of each string was tied onto both sides of the midpoint of the bottom of the cage 13 cm apart. The seven tasks (T1–T7) were structured as follows: T1, the task of parallel strings, in which two 25-cm long parallel strings were set perpendicular to the bottom of the cage. The free end of one string was connected to a bait and the free end of the other string was empty; T2, the task of slanted strings, with two 25-cm long parallel strings forming a 45°-angle to the bottom of the cage and the bait closer to the middle point between the two strings; T3, the task of slanted strings, with the bait farther from the middle point between the two strings, similar to T2 but with the free end of the string connected to a bait located near the center of the cage; T4, the task of long-short strings, in which two parallel strings (one 30-cm long and the other 15-cm long) were placed perpendicular to the bottom of the cage and the two free ends were connected to a bait; T5, the task with two crossing strings, in which two 25-cm long strings intersected at 90°, with the free end of one string connected to a bait and the other being empty; T6, the task with a right-angled turn on the longer baited string and two parallel strings were placed perpendicular to the bottom of the cage (one 20-cm long and the other 40-cm long). The free end of the 20-cm string was empty and the 40-cm long end was connected to a bait but at a right angle to the other string, 25-cm from the bottom of the cage; and T7, the task of continuity and discontinuity, in which two 25-cm long parallel strings were placed perpendicular to the bottom of the cage and each of the two free ends were connected to a bait; one of the two strings was disconnected at 5 cm, 15-cm from the cage. The tasks from T1 to T7 in this study were arranged in the order from easy to difficult.

Parallel strings (T1) test the means-end understanding goal directedness; slanted (T2 and T3), long-short (T4), cross (T5) and turning (T6) strings the proximity principle; and disconnected strings (T7) whether the subjects can understand the continuous/discontinuous nature of strings (Hofmann, Cheke & Clayton, 2016).

### Data analysis

Data were tested using the two-tailed binominal test (SPSS 23.0, Chicago, IL, USA), with *p* values at 0.05 for significance, 0.01 for extremely significance, or 0.001 for highly extremely significance (Tables 2 and 3). Lateral bias index (LBI) was used to analyze the direction and intensity of the side bias to the strings by individual oriental magpies (Damerose & Hopkins, 2002). This was achieved by calculating the ratio of the number difference (R − L) between the right sided selection (R) and the left (L) over the sum of the two selections (R + L), that is, LBI = (R − L)/(R + L). The LBI score ranged from −1.0 to 1.0; when the score was less than 0, the left side was preferred, otherwise the right was preferred. The absolute value of the LBI score (referred to as ABS-LBI) showed the side-bias intensity of the tested individuals.

**Table 2:** Degrees in solving the seven tested tasks.

	T1			T2		T3		T4		T5		T6		T7	
	*C*	*p*	*C*		*p*	*C*	*p*	*C*	*p*	*C*	*p*	*C*	*p*	*C*	*p*
P1	19	0.200		13	0.585	–	–	–	–	10	0.099	18	0.362	19	0.200
P2	15	1.000		13	0.585	20	0.099	17	0.585	12	0.362	16	0.856	14	0.856
P3	12	0.362		12	0.362	–	–	–	–	18	0.362	14	0.856	14	0.856
P4	17	0.585		16	0.856	–	–	–	–	14	0.856	16	0.856	14	0.856
P5	27***	<0.001		9*	0.043	19	0.200	–	–	9*	0.043	17	0.585	18	0.362
P6	19	0.200		12	0.362	26***	<0.001	22*	0.016	13	0.585	13	0.585	–	–
P7	18	0.362		10	0.099	–	–	–	–	17	0.585	11	0.200	17	0.585
P8	19	0.200		4***	<0.001	19	0.200	18 (26)	0.076	10	0.099	14	0.856	11	0.200

**Notes:**

Significant differences from random choice (Two-tailed binomial test): *p < 0.05; **p < 0.01; ***p < 0.001. T1–T7, Task 1 through Task 7; P1–P8, oriental magpies P1–P8; C, number of correct trials; “–”, tests failed due to the individual bird was unwilling to participate in the task. Results showed were the correct times during 30 trials except in one case of 26 trials (in brackets).

**Table 3:** Bird’s side bias strategies for choosing the direction of the string.

	T1			T2			T3			T4			T5			T6			T7		
	*L*	LBI	*p*	*L*	LBI	*p*	*L*	LBI	*p*	*L*	LBI	*p*	*L*	LBI	*p*	*L*	LBI	*p*	*L*	LBI	*p*
P1	17	−0.13	0.585	11	0.27	0.200	–	–	–	–	–	–	15	0	1.000	12	0.20	0.362	20	−0.33	0.099
P2	26	−0.73***	<0.001	23	−0.53**	0.005	23	−0.53**	0.005	15	0	1.000	23	−0.53**	0.005	17	−0.13	0.585	27	−0.80***	<0.001
P3	27	−0.80***	<0.001	24	−0.60***	<0.001	–	–	–	–	–	–	5	0.67***	<0.001	28	−0.87***	<0.001	29	−0.93***	<0.001
P4	16	−0.07	0.856	25	−0.67***	<0.001	–	–	–	–	–	–	27	−0.80***	<0.001	23	−0.53**	0.005	29	−0.93***	<0.001
P5	14	0.07	0.856	20	−0.33	0.099	20	−0.33	0.099	–	–	–	18	−0.20	0.362	25	−0.67***	<0.001	24	−0.60***	<0.001
P6	24	−0.60***	<0.001	11	0.27	0.200	13	0.13	0.585	8	0.47*	0.016	18	−0.20	0.362	2	0.87***	<0.001	–	–	–
P7	19	−0.27	0.200	14	0.07	0.856	–	–	–	–	–	–	2	0.87***	<0.001	8	0.47*	0.016	3	0.80***	<0.001
P8	12	0.20	0.362	15	0	1.000	17	−0.13	0.585	16 (26)	−0.23	0.327	14	0.07	0.856	26	−0.73***	<0.001	26	−0.73***	<0.001

**Notes:**

L, side bias for the left strings during 30 trials except in one case of 26 trials for P8; LBI, lateral bias index, which ranges from −1.0 to 1.0. If the value of LBI is less than 0, the left side is preferred, otherwise, the right side is preferred; *p*, *p*-value. Significant differences in two-tailed binomial test: *p < 0.05; **p < 0.01; ***p < 0.001. T1–T7, Task 1 through Task 7; P1–P8, oriental magpies P1 through P8. “–”, tests failed due to the individual bird was unwilling to participate in or did not complete the task.

### Ethical approval

The rearing of birds strictly complied with the requirements of “Animal Feeding Standards of the Beijing Wildlife Rescue Center.” Animal handling during testing was conducted in accordance with the requirements of the “Animal Ethics Committee of Beijing Forestry University.” All applicable international, national and institutional guidelines for the care and use of animals were followed. Under Chinese law, no specific approval was required for this noninvasive study. This article does not contain any studies with human participants.

## Results

### Pre-testing and training success of the individual birds

Only one of the 11 oriental magpies familiar with the experimental environment (P6) could pull a string spontaneously to get bait without signs of stress or neophobia (T1; Fig. 3; Table 1). P6 attempted to pull the string and retrieve the bait the first time it faced the pulling task and took only 37 s from start of the experiment. Seven magpie individuals (P1–P5, P7 and P8) passed the pre-testing after learning and training. Four birds (P2–P5) passed during the learning phase and three (P1, P7 and P8) passed with training after previous learning failure. An oriental magpie (P3) in the first stage of the training accidentally pulled a string with one of its claws and found that it could get bait by pulling; this individual was classified as learning to solve the string-pulling task. Eight individuals (P1–P8) were subjected to the second stage of trials to solve the tasks shown in Fig. 3. The other three birds did not properly participate in the experiment at any point and thus were not listed in Table 1.

### Tasks with parallel strings

P5 successfully completed the parallel strings task (T1; Fig. 3) (Accuracy Rate (AR) = 90.0%, significant differences of two-tailed binomial test: *p* < 0.001; Table 2). P2, P3 and P6 were among the individuals that could not solve the parallel task but showed a significant side bias for the left side of the two strings (*p* < 0.001; Table 3).

### Tasks with slanted strings

None of the eight birds were able to solve the task of slanted strings with the bait closer to the middle point between the two strings (T2; Fig. 3), and P5 and P8 showed significantly low accuracy (P5: AR = 30.0%, *p* = 0.043; P8: AR = 13.3%, *p* < 0.001; Table 2). In addition, P2 (*p* = 0.005; Table 3), P3 and P4 (*p* < 0.001) showed a significant side bias for the left side of the two strings. P6 successfully completed the task (AR = 86.7%, *p* < 0.001) of slanted strings with the bait closer to the middle of the two strings (T3; Fig. 3), but P1, P3, P4 and P7 were reluctant to participate in the task (Table 2). P2 showed a significant side bias for the left string during the test (*p* = 0.005; Table 3).

### Task with long-short strings

P2, P6 and P8 were the only subjects with a willingness to try to solve the task of long-short strings (T4, Fig. 3). P6 preferred the short string (AR = 73.3%, *p* = 0.016), while P2 (AR = 56.7%; *p* = 0.585) and P8 (AR = 69.2%; *p* = 0.076) had no significant preference for the long or short string. P6 also had a significant side bias to the left string (*p* = 0.016; Table 3).

### Task with two crossing strings

None of the eight birds could solve the task with two crossing strings (T5; Fig. 3) and the accuracy rates of P1, P5 and P8 were relatively low (P1 and P8: AR = 33.3%, *p* = 0.099; P5: AR = 30%, *p* = 0.043; Table 2). P2 (*p* = 0.005; Table 3) and P4 (*p* < 0.001) showed significant side bias for the left string and P3 and P7 were biased toward the right string during the test (*p* < 0.001).

### Tasks with turning and continuity strings

In the task with a right-angled turn on the longer baited string (T6; Fig. 3) and the task of continuity and discontinuity (T7; Fig. 3), all subjects attempted the two tasks, except P6 who was unwilling to attempt T7. The birds showed no ability to solve either task (Table 2). There was a significant left-sided bias among P3, P4, P5 and P8 during T6, and P2, P3, P4, P5 and P8 during T7 (*p* < 0.001; Table 3), whereas there was a right-side bias by P6 (*p* < 0.001) and P7 (*p* = 0.016) during T6 and P7 during T7 (*p* < 0.001).

## Discussion

Our study revealed one female oriental magpie that spontaneously passed the pre-testing, and seven (four males and three females) that learned to obtain food by pulling a string after a period of learning and/or training (Table 1) during the first stage of the experiment. These eight birds participated in the second stage of the experiment with the seven baited multiple-string problems (T1–T7; Fig. 3), of which only P5 solved the parallel task (T1; Table 2), and P6 solved one of the two tasks of slanted strings (T3) without learning. Part of our results were similar to those from reports on western scrub-jay (*Aphelocoma californica*) (Hofmann, Cheke & Clayton, 2016), the common raven (*Coravus corax*), and the hooded crow (*C*. *cornix*) (Bagotskaya, Smirnova & Zorina, 2012; Obozova et al., 2014). The number of tests conducted by different researchers was different. For examples, the raven and western scrub-jay were tested 32 and 50 times in each of their tasks, respectively, and the hooded crow 30–32 times in tasks of one report and 20 times in tasks of another (Bagotskaya, Smirnova & Zorina, 2012; Obozova et al., 2014; Hofmann, Cheke & Clayton, 2016). So we only compared the binomial test results of our magpie and other birds in the family Corvidae who also performed horizontal string-pulling tasks. Three of four common raven solved tasks like our T1 and all the four raven solved those like T3, three of four hooded crows solved those like T1 and T3, and four of five western scrub-jay solved the task like T1 and all the five western scrub-jay solved that like T3 (Bagotskaya, Smirnova & Zorina, 2012; Hofmann, Cheke & Clayton, 2016). In the above three bird species, the ratios of success for individuals in participating the tasks like our T1 and T3 were higher; but only two of our magpies solved the two tasks (i.e., P5 solved T1 and P6 solved T3). There were individuals in raven (one of four), hooded crow (two of four), and western scrub-jay (one of five) solving the task like T2 of this study, whereas all magpies of this study could not solve T2 and two magpies (P5 and P8) showed significant proximity principle. Our magpie was similar to the hooded crow, they couldn’t solve the cross string (T5) and showed significant proximity principle (two of eight in two researches respectively), and there was also one magpie (P6) showing a significant proximity principle (Bagotskaya, Smirnova & Zorina, 2012; Obozova et al., 2014). The hooded crow could solve both tasks like our T6 (four of the eight crows) and T7 (continuity strings; six of eight crows), but only one of our magpies (P1) could solve the task of turning (T6) through trial and error learning, and all of them couldn’t solve the task of continuity strings (Bagotskaya, Smirnova & Zorina, 2012; Obozova et al., 2014). In a task like our T6, one hooded crows showed significantly proximity principle, but we did not find our magpies used proximity principle to solve the task of turning strings (Bagotskaya, Smirnova & Zorina, 2012).

Subjects in our study could not solve one of the slanted strings tasks (T2; Fig. 3; Table 2) or the cross string task (T5; Fig. 3) as the common raven could, and the task of continuity and discontinuity (T7) as hooded crow did (Bagotskaya, Smirnova & Zorina, 2012; Obozova et al., 2014). The eight magpies in this study solved fewer multiple-string tasks than the *Corvus* birds (only three out of eight magpies solved a few multiple-string problems) despite their close evolutionary relationship (Ericson et al., 2005). When faced the tasks like T2 and T5, some individuals (e.g., P5 and P8) showed significant selection errors (*p* = 0.043 and *p* < 0.001, respectively, Table 2). P8 did not employ a significant selection strategy of the proximity principle in the long-short tasks (T4). The same bird seemed to adopt different selection strategies when faced with different tasks.

Among the three oriental magpies (P2, P6 and P8) participating in the task of long-short strings (T4; Fig. 3), one subject (P6) showed an obvious preference to the short string side (*p* = 0.016; Table 2). In the study of western scrub-jay, all the five birds had no preference in the tasks of first 50 times, but all of them preferred short-term tasks in the last 50 tasks, which was different from the results of our study (Hofmann, Cheke & Clayton, 2016). In other studies, the bait was maintained at a fixed distance to the cage wall with strings of different lengths (i.e., one of the strings was curved). The birds were unable to solve this string problem, although they exhibited behaviors by the principle of proximity. These phenomena might be due to the fact that the bird recognized that it could get access to the bait only by pulling the string nearer to bait (i.e., the proximity principle), rather than truly understanding the combined structure and relationship between the string and bait. This reaction may be explained by the perceptual-motor feedback loop rather than a comprehension of the means-end relation of string and reward (Hofmann, Cheke & Clayton, 2016).

We analyzed the change of accuracy times of individual magpie in each task, and found that P1 and P5 had trial and error learning behavior in solving multiple-string problem (T1 and T6) after excluding the individuals with side bias strategy. P1 used the strategies of preference and trial-and-error learning during the first 3 days in solving the task with a right-angled turn on the longer baited string (T6, right five times in 14 trials, *p* = 0.424; Fig. 3). This was followed by an increase of its string pulling AR over the next two days, with 13 out of the 16 tests being successful (AR = 81.3%, *p* = 0.021). The AR of P5 in T1 increased with time (77.8%, 87.5%, 100%, and 100% on day 1–4, respectively), and the AR of P1 was not stable, but on the last day it has been increased significantly (P1: 60%, 50%, 80% on day 1–3, respectively). The AR of P6 was also not stable, which increased with time in 1–2 days of T1 test (P6: 60%, 80%, 50% on day 1–3, respectively), but P6 showed strong left side bias in the third day’s trials (left string selected in 10 tests). In addition, we found no other magpies that increased the accuracy rate through trial and error learning. This result indicates that by trial and error learning, oriental magpies might be able to solve certain multiple-string tasks. However, the oriental magpie’s solution to the task may not be based on the understanding of the relationship between the strings and bait but rather is an accumulation of learning and experience (Seibt & Wickler, 2006; Taylor, Knaebe & Gray, 2012). The subjects may understand that the string is a means to reach a goal, but do not understand the mechanism of connectedness (Jacobs & Osvath, 2015).

The bait appeared randomly at the end of either the left and/or the right sides of the strings in our study. P5 and P6 solved one- and two-multiple-string problems, respectively (Table 2). However, the other six birds that did not completely solve any tasks, similar to common raven reported by Heinrich (1995), showed different simple strategies such as side bias strategies (i.e., to choose only one side of the string, regardless of whether the string was connected to a bait), trial-and-error learning, the proximity principle, and random selection. Most of the magpies ultimately did not acquire the ability to solve any of the tasks despite showing certain trial-and-error learning behavior after choosing the wrong string, which is in contrast to results from the kea parrot (*Nestor notabilis*) in its pulling experiments (Werdenich & Huber, 2006). P1 was the exception and completed the right angle turn task (T6) through trial-and-error learning.

Although our magpies made their choice after a certain period of observation, they still could not solve most of the multiple-string problems. Only when the baits are displaced could they realize whether the chosen string is correct. This further illustrates that the magpie’s pulling behavior was based on the perceptual-motion feedback loop, rather than understanding the means-end relation of string and reward. The correct choice should have been visually obvious without having to pull the string first and the magpies should have noticed an incorrect string choice and adapted their strategy. Hofmann, Cheke & Clayton (2016) considered this awareness of errors and adjustment of strategies as precursors to the physical problem to be solved, providing a basis for the development of causal understanding. However, each bird was tested only 30 times for each task, limiting our ability to determine whether each magpie could eventually solve the given tasks through learning or other strategies. Nevertheless, one magpie showed the ability to solve the task by changing strategies and through trial-and-error learning in the right-angle string task (T6; Fig. 3), suggesting that certain individuals of this species can learn to solve multi-string problems. However, further study is required to determine whether the oriental magpie can understand the causal mechanism behind multi-string tasks.

## Conclusions

Oriental magpies used learning and training to understand that pulling strings gave them access to bait. As a result, two magpies solved several tasks without prior exposure to multiple string tasks before onset of the experiment (Table 2). In addition, one magpie solved the task of turning string (T6) through trial and error learning. However, they were not able to solve more complex tasks, such as two crossing strings (T5; Fig. 3) and the task of continuity and discontinuity (T7). When they faced of the problems which they could not solve, different individuals showed different strategies, such as proximity principle, side bias strategies, random selection, and trial and error learning. It may be suggested that the overall cognitive ability of the oriental magpie species used in this study is poorer than that of the large birds in the family Corvidae, especially *Corvus* species. Crows may have evolved superior intelligence owing to their complex and changeable environment (Seed, Emery & Clayton, 2009). The oriental magpie is no exception, but they failed to solve more problems because they lacked experience in solving multi-string tasks (Taylor et al., 2010). It should be noted that only eight magpies were tested in this study, thus the conclusions may be limited.

## Supplemental Information

10.7717/peerj.9200/supp-1 Supplemental Information 1 Raw Data of Oriental Magpie String-pulling Task. Oriental magpie individuals (P1–P8) solving the multiple-strings tasks (T1–T7).

## Additional Information and Declarations

### Data Availability

The following information was supplied regarding data availability: The raw data for the Oriental magpie individuals in solving the multiple-strings tasks (T1–T7) are available as a Supplemental File.
